# N-Alpha-Acetyltransferases and Regulation of CFTR Expression

**DOI:** 10.1371/journal.pone.0155430

**Published:** 2016-05-16

**Authors:** Ali J. Vetter, Andrey L. Karamyshev, Anna E. Patrick, Henry Hudson, Philip J. Thomas

**Affiliations:** Department of Physiology, University of Texas Southwestern Medical Center, Dallas, Texas, United States of America; The Ohio State University, UNITED STATES

## Abstract

The majority of cystic fibrosis (CF)-causing mutations in the cystic fibrosis transmembrane conductance regulator (CFTR) lead to the misfolding, mistrafficking, and degradation of the mutant protein. Inhibition of degradation does not effectively increase the amount of trafficking competent CFTR, but typically leads to increased ER retention of misfolded forms. Thus, the initial off pathway steps occur early in the processing of the protein. To identify proteins that interact with these early forms of CFTR, *in vitro* crosslink experiments identified cotranslational partners of the nascent chain of the severe misfolded mutant, G85E CFTR. The mutant preferentially interacts with a subunit of an N-alpha-acetyltransferase A. Based on recent reports that acetylation of the N-termini of some N-end rule substrates control their ubiquitination and subsequent degradation, a potential role for this modification in regulation of CFTR expression was assessed. Knockdown experiments identified two complexes, which affect G85E CFTR proteins levels, NatA and NatB. Effects of the knockdowns on mRNA levels, translation rates, and degradation rates established that the two complexes regulate G85E CFTR through two separate mechanisms. NatA acts indirectly by regulating transcription levels and NatB acts through a previously identified, but incompletely understood posttranslational mechanism. This regulation did not effect trafficking of G85E CFTR, which remains retained in the ER, nor did it alter the degradation rate of CFTR. A mutation predicted to inhibit N-terminal acetylation of CFTR, Q2P, was without effect, suggesting neither system acts directly on CFTR. These results contradict the prediction that N-terminal acetylation of CFTR determines its fitness as a proteasome substrate, but rather NatB plays a role in the conformational maturation of CFTR in the ER through actions on an unidentified protein.

## Introduction

Cystic fibrosis (CF) is an autosomal recessive disorder that affects the function of many organs (pancreas, lungs, sweat glands) due to the loss of a chloride channel, the cystic fibrosis transmembrane conductance regulator (CFTR), located in the apical membrane of epithelial cells. There are hundreds of mutations found in the CFTR gene that are associated with disease; classified into four or more different molecular pathology groups [1]. A recent study determined that of ~40,000 CF patients, 159 different CFTR variants accounted for 96% of the alleles [2]. Of these variants, 133 were classified into one of the four CF-disease causing groups [1, 2]. The most prevalent class (Class II) contains mutations that cause the misfolding and thereby the mistrafficking of CFTR. Misfolded CFTR is typically retained in the endoplasmic reticulum (ER) and subsequently degraded by the proteasome through the endoplasmic reticulum-associated degradation pathway (ERAD).

The conformational maturation of CFTR in the ER is complex and incompletely understood. Some early studies determined that there are two forms of ER retained CFTR [3, 4]. The major fraction is a proteasome-sensitive form destined for degradation; while a smaller fraction is a proteasome-insensitive and ER export competent form whose formation is dependent on ATP [3]. These multiple forms of CFTR in the ER reside in distinct subER locations [5, 6]. The ER export competent is trafficked from the ER to the Golgi by COPII, if CFTR interacts with the recognition protein of the COPII machinery, Sec24c [6]. Unfortunately, inhibition of the proteasome does not improve trafficking of mutant CFTR to the plasma membrane, but rather leads to an accumulation of the protein in the ER [7] indicating that the proteasome-sensitive form of CFTR is not in equilibrium with the productive folding pathway. To further understand the maturation process of CFTR, this study examined early protein interactions with the nascent chain of CFTR during translation.

For other substrates, nascent chain-protein interactions at the ribosome act as an early quality control mechanism. One such group of protein complexes are the N-alpha-acetyltransferases; enzyme complexes involved in the cotranslational N-terminal acetylation of substrates. They catalyze the transfer of an acetyl group from acetyl coenzyme A (acetyl-CoA) to the NH_3+_ group on the substrate [8]. The complex consists of a catalytic subunit, which is bound to the ribosome in proximity to the nascent chain by auxiliary subunits. The most abundant N-alpha-acetyltransferase complex in mammalian cells is NatA. NAA16 and NAA15 are auxiliary subunits that interact with the same catalytic subunit, NAA10, to form two distinct dimeric NatA complexes. Previous work indicates that NatA acetylates substrates after the removal of the initiator methionine residue [8]. NAA15 and NAA10 can also interact with another catalytic subunit NAA50 to form the trimeric NatE complex, which acetylates Met-Leu- and Met-Ile- N-termini [8]. CFTR’s N-terminus (Met-Gln) is predicted to be acetylated by NatB, a complex composed of the catalytic subunit NAA20 and auxiliary subunit NAA25 [8].

N-terminal acetylation has been implicated in regulating the stability of proteins by either creating a degron signal for ubiquitin ligase recruitment [9] or, conversely, by protecting the N-terminus from being ubiquinated [10]. The goal of this study was to determine if mutations in CFTR lead to differential interactions with ribosome-associated proteins, such as Nat complexes, and whether such interactions play a role in the preferential degradation or conversion of the proteasome-sensitive form of ER-retained CFTR to the ER export competent form.

## Results

### Identification of Proteins Interacting with the Nascent Chain of CFTR

**Fig 1:**
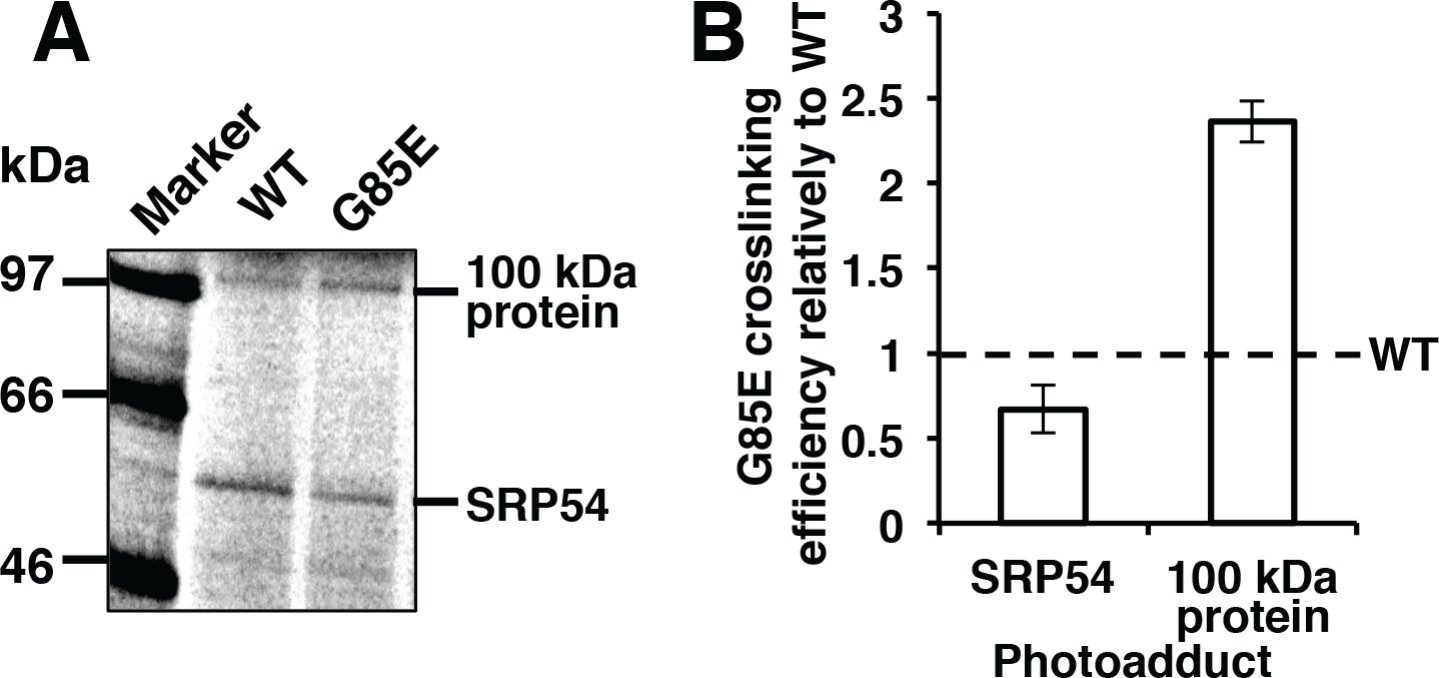
Early Co-translational Proximity of CFTR. (A) Radiograph of crosslinking products formed with WT and G85E CFTR *in vitro* translated in wheat germ lysate after separation by SDS-PAGE. (B) Quantitative analysis of the photoadducts. Error bars are SEM. Band intensity measurements conducted by the use of ImageJ software.

In order to determine protein-nascent chain interactions during the earliest steps in CFTR maturation, *in vitro* translation experiments comparing WT and G85E containing a modified amino acid with a photoactivatable crosslinking moiety at position 93, located in transmembrane span 1 and near their N-termini, were conducted in a wheat germ system. Truncated CFTR mRNAs lacking a natural stop codon generated radiolabeled nascent chains length of 156 codons that remain associated with the ribosome with approximately 126 residues exposed from the exit tunnel. Fig 1A illustrates a representative result of crosslink products formed on the translating ribosome. One of the photoadducts was identified as a known binding partner of CFTR transmembrane spans, SRP54. An additional unknown binding partner produced a photoadduct suggesting it is a protein of 100 kDa molecular weight (Fig 1A). Comparison of WT and G85E CFTR reveal a reduction in the interaction of the mutant with SRP54 and a concomitant increase in crosslinking to the 100 kDa protein (Fig 1B). These results suggest this protein plays a role in the recognition of mutated CFTR by cellular quality control machinery.

**Fig 2:**
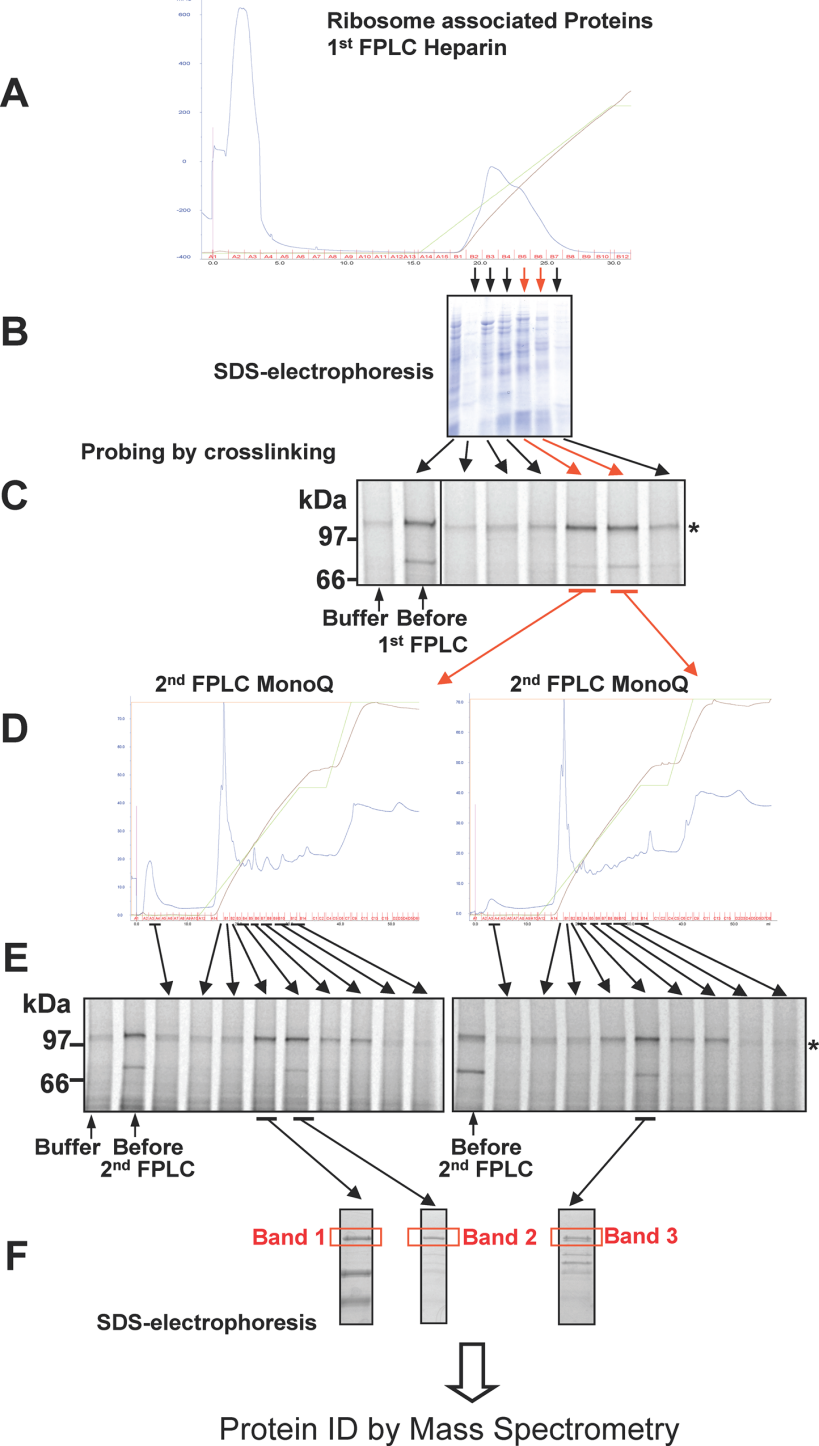
Scheme for the Identification of Proteins Interacting with CFTR Nascent Chains During Translation. Ribosome-associated proteins were released from wheat germ ribosomes by high salt treatment and were subjected to dialysis. Proteins were fractionated by FPLC on Heparin column (A), fractions were tested by crosslinking analysis (C), and active fractions were subfractionated by FPLC on MonoQ column (D), and tested by crosslinking analysis again (E), proteins in the active subfractions were separated by SDS-PAGE (F), and bands of appropriate molecular weight were excised from the gel and analyzed by mass spectrometry. B, Electrophoresis of fractions (Coomassie).

To identify the interacting protein, they were stripped from the ribosome nascent chain complexes by high salt wash and fractions of the desalted wash were used to reconstitute the photoadduct formation. In the current work, the ribosome-associated proteins were fractionated by FPLC on a Heparin column (Fig 2A and 2B) and fractions containing ~100kDa protein identified by photocrosslinking. Fractions were further separated by FPLC MonoQ column (Fig 2D) and again, subfractions enriched in ~100kDa proteins were selected based on the photocrosslinking assay (Fig 2E). SDS-electrophoresis assessed the complexity of these fractions (Fig 2F). The protein bands corresponding to ~100kDa and surrounding areas (from three different fractions containing the protein of interest) were excised from the gel and analyzed by mass spectrometry. This approach for identification of proteins interacting with nascent chains has been named iPINCH [11]. The approach identified the unknown 100 kDa protein as a plant protein (S1 Table, Bands 1–3) with the closest human homologues being N-alpha-acetyltransferase 16 (NAA16) and NAA15 (identified by BLAST, not shown). An alternate scheme for fractionation and protein identification produced nearly identical results (S1 Fig, S1 Table, Band 4) Due to the increased proximity of NAA16/15 with a Class II mutant form of CFTR, G85E, the following hypothesis was formulated: Decreased interaction with SRP54 allows increased N-terminal acetylation of G85E CFTR by NatA produces a degron signal, which is efficiently degraded by the proteasome. By contrast, WT CFTR’s efficient interaction with SRP54 interferes with acetylation while promoting proper maturation of the protein.

### N-Alpha-Acetyltransferases and Steady State CFTR G85E Protein Levels

**Fig 3:**
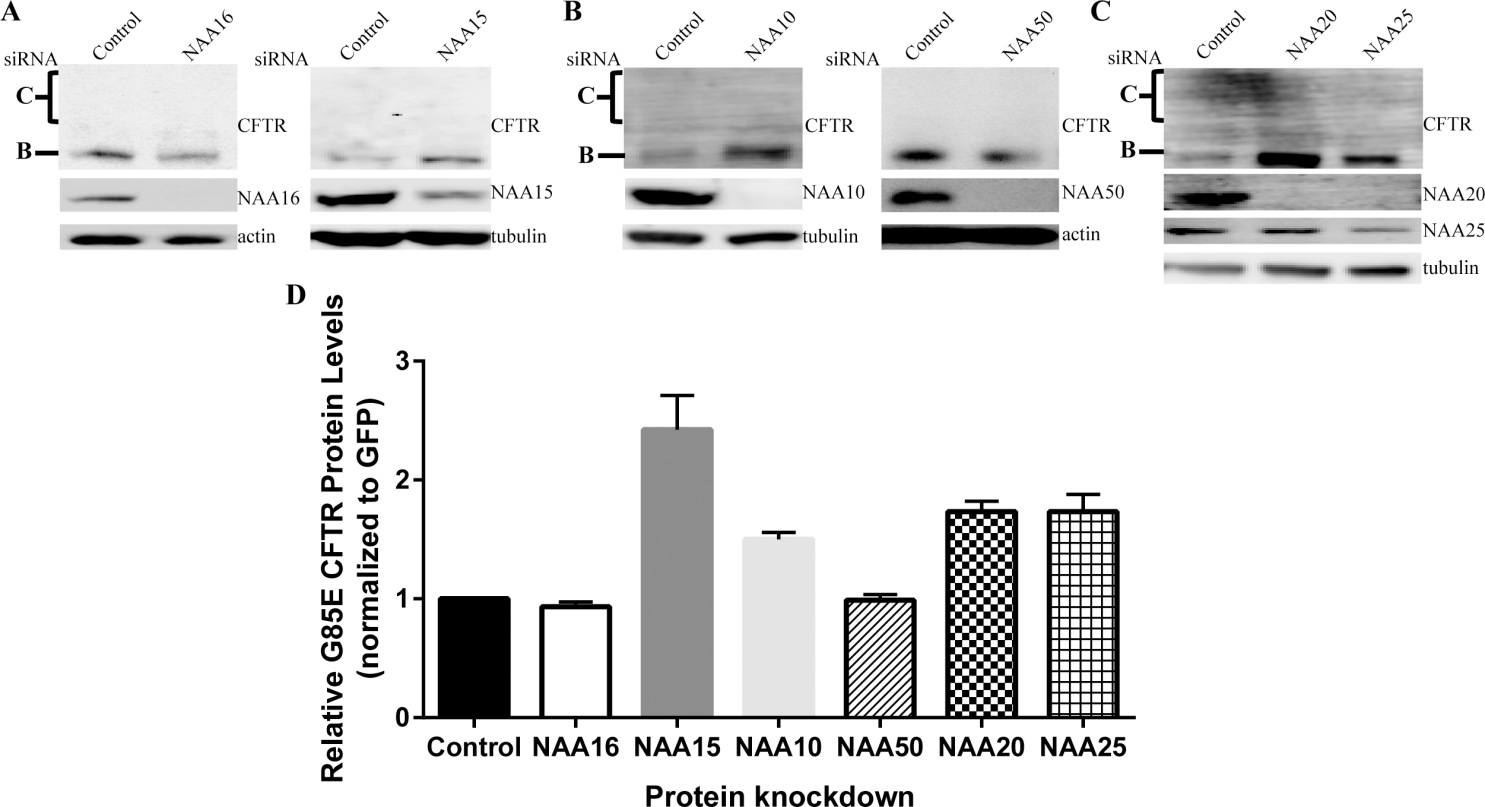
Effects of NatA, NatE, and NatB Complex on G85E CFTR Protein Levels. Representative Western blots after SDS PAGE of steady state protein levels of G85E CFTR in cultured HeLa-Tet-On cells upon depletion of A) NAA16 (NatA), NAA15 (NatA/E), B) NAA10 (NatA), NAA50 (NatE), C) NAA20 (NatB), or NAA25 (NatB)). Actin or tubulin are loading controls. D) Quantitation by LiCor and statistics (n = 3, +/- SEM).

The first NAAs examined for involvement with G85E CFTR were the homologues of the auxiliary subunit shown to be in proximity to the CFTR nascent chain by the crosslink experiments, human NAA15 and NAA16. In order to determine if either subunit affected G85E CFTR protein levels, siRNA was used to knockdown NAA15 and NAA16 in HeLa-Tet-on cells (Fig 3A). Despite a dramatic decrease in NAA16 protein levels, G85E CFTR levels remained unchanged (Fig 3A and 3D) indicating that NAA16 activity is not relevant to CFTR expression. By contrast, knockdown of NAA15, led to a 2.5-fold increase in steady state G85E protein levels compared to control levels (Fig 3A and 3D). Thus, even though both NAA15 and NAA16 are capable of forming a complex with the same catalytic subunit [8], NAA10, there is specificity for NAA15. Whereas NAA15 can act as an auxiliary subunit for both the NAA10 (NatA) and NAA50 (NatE) catalytic subunits; the specificity for the catalytic subunit was next examined. Knockdown experiments showed that only reduction of NAA10 affected G85E CFTR levels with a 1.5-fold increase compared to control (Fig 3B and 3D). Similar to NAA16, G85E CFTR protein levels were unchanged upon knockdown of NAA50 (Fig 3B and 3D) ruling out an effect of NatE. These results establish specificity in the regulation of G85E CFTR for NatA consisting of NAA10-NAA15 subunits.

However, the N-terminal sequence of CFTR is not predicted to be a substrate for NatA [8]. The complex NatB, composed of the catalytic subunit NAA20 and the auxiliary subunit NAA25, is predicted to acetylate Met-Gln- N-termini like that of CFTR [8]. Knockdown of either the catalytic subunit, NAA20, or the auxiliary subunit, NAA25, increased G85E CFTR protein levels 1.8 fold compared to control (Fig 3C and 3D). Taken together, these results indicate that G85E CFTR protein levels are regulated by the NatA isoform composed of NAA10-NAA15, and by NatB composed of NAA20 and NAA25; but not by the NatA isoform containing NAA10-NAA16 nor by NatE composed of NAA50-NAA10-NAA15.

### Role of the N-Terminus of CFTR in NatA/B Regulation

**Fig 4:**
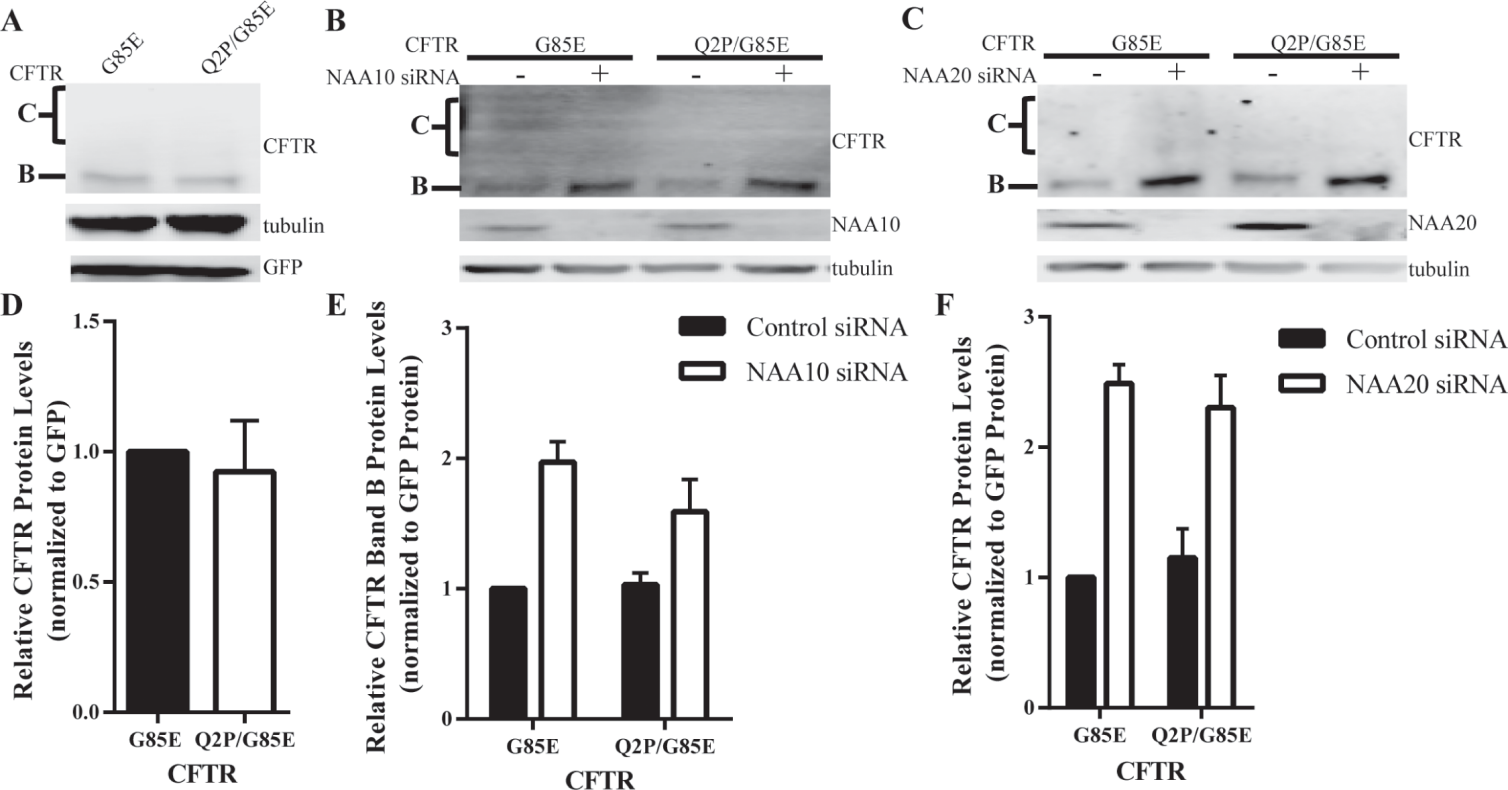
Requirement for CFTR’s N-terminus in NatA/B Regulation. Steady state protein levels of CFTR in cultured HeLa-Tet-On cells expressing the Q2P mutation (A) as well as after depletion of NAA10 (B), or NAA20 (C) were monitored by Western blotting after separation of cell lysate by SDS-PAGE. Representative Western blots presented (A-C). Tubulin was a loading control. D-F) Quantitation and statistical analysis (n = 3, +/- SEM).

All residues found at the N-terminus have the possibility of being acetylated by one of the six NAA complexes, except when the second residue is a proline [12, 13]. To assess whether the activity of NatA or NatB to regulate CFTR levels depends upon direct acetylation of CFTR’s N-terminus, a proline, which prevents all known N-terminal acetylation reactions, was introduced [12, 13] in place of the native glutamine at position 2 of CFTR, Q2P, on the G85E CFTR background. If direct acetylation of CFTR by NatA or NatB were responsible for the decrease in CFTR levels relieved upon knockdown of the transferase, the Q2P mutant would also be predicted to lead to an increase in steady state levels of CFTR. Contrary to the prediction, when expressed in HeLa-Tet-On cells, Q2P/G85E CFTR protein levels were similar to wild-type G85E CFTR (Fig 4A and 4D) indicating that direct acetylation of CFTR by either of these or any known transferase complexes is not responsible for alteration in its expression. Moreover, the Q2P/G85E CFTR mutant protein levels were examined after knockdown of NatA and NatB confirming that the acetylation of CFTR’s N-terminus was not required for their effect on G85E CFTR expression. Reduction of either complex continued to cause an increase of 2-fold compared to control conditions (Fig 4B, 4C, 4E and 4F). Taken together, these results indicate that CFTR is not directly N-terminal acetylated and thus, N-terminal acetylation of CFTR itself cannot explain the increase in steady state levels of CFTR protein in response to reduction of NatA or NatB. The increased proximity of NAA15/16 with the G85E nascent chain demonstrated in the crosslinking experiments does not reflect direct acetylation of the N-terminus of CFTR, but rather suggest that NatA and NatB effects on G85E CFTR levels must occur through an allosteric pathway.

### NatA/B and the Degradation of G85E CFTR

**Fig 5:**
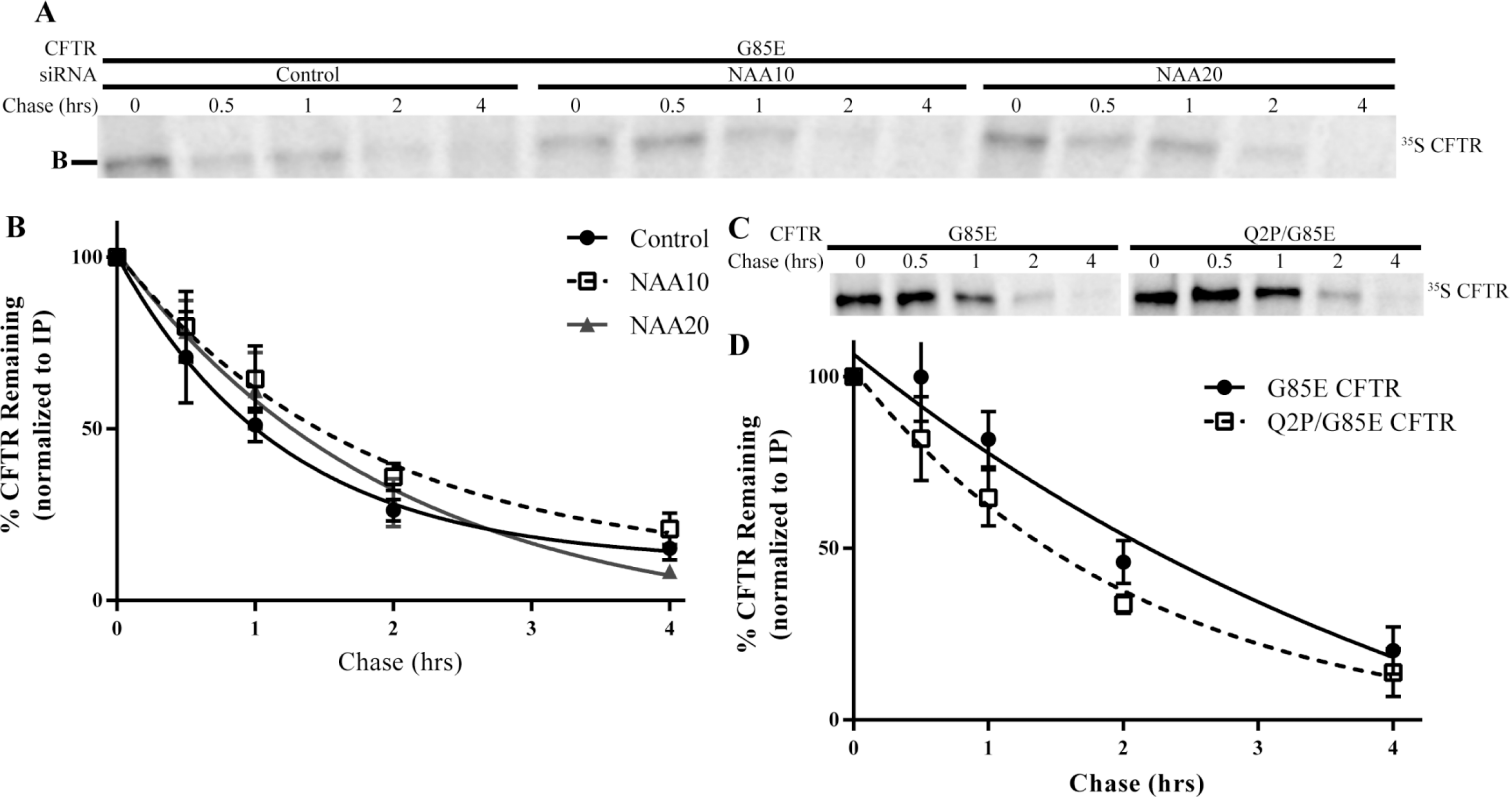
Degradation Rates of G85E CFTR. Radiographs of SDS-PAGE of immunoprecipated (IP) A) G85E CFTR from cell lysates depleted for NAA10, NAA20, or for c) the G85E/ Q2P CFTR double mutant after a thirty-minute pulse and subsequent chase for the times indicated. Quantification and statistical analysis of radiograph signals (n = 6, +/- SEM) for panel A are in B) and for panel C) in D).

Protein expression can be controlled by regulation of transcription, mRNA stability, translation or protein degradation. Notably, N-terminal acetylation has been implicated in many of these processes. The hypothesis initially tested in this work was based on the role of acetylation in regulating the degradation of proteins [9, 10]. In order to determine whether NatA or NatB activity regulate G85E CFTR degradation rates, pulse-chase experiments were performed with and without knockdown of NatA or NatB (Fig 5). The half-life of G85E CFTR under all three conditions was determined from the rate of degradation after a thirty-minute pulse labeling: The half-lives were, control = 50 +/- 20 mins, knockdown of NAA10 = 74 +/- 22 mins, and knockdown of NAA20 = 84 +/- 22 mins (Fig 5A and 5B). Thus, none of the determined half-lives was statistically different under any of the conditions (Fig 5B) suggesting that NatA and NatB do not regulate steady state protein levels through the degradation of CFTR. Similarly, the degradation rate of the Q2P/G85E CFTR was also within statistical error of G85E CFTR (G85E = 100+/- 20 mins and Q2P/G85E = 85 +/- 21 mins) (Fig 5C and 5D) indicating that the changes in steady state levels of CFTR observed are inconsistent with the hypothesis that N-terminal acetylation of CFTR produces a degron.

### mRNA Levels of G85E CFTR

**Fig 6:**
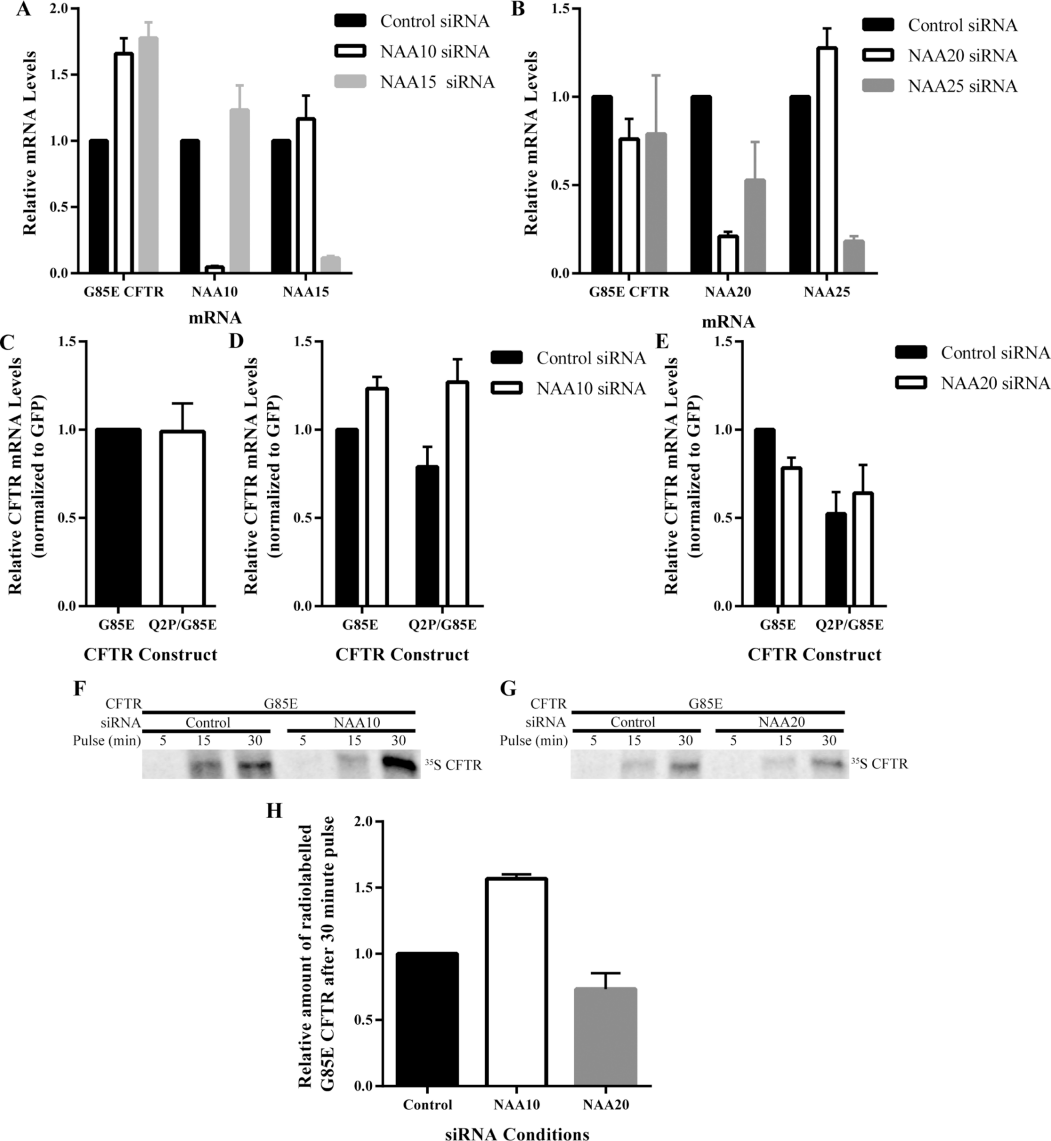
Effect of NatA/B on mRNA Levels and Translation Rates of G85E CFTR. RT qPCR analysis of G85E CFTR, NAA10, and NAA15 mRNA levels after RNA purification of cell lysates (n = 4, +/- SEM) depleted for A) NatA: NAA10and NAA15, or B) NatB: NAA20 and NAA25. RT qPCR analysis of G85E and Q2P/G85E CFTR mRNA levels after RNA purification from cell lysates (n = 4, +/- SEM) from C) control cells or cells depleted of D) NAA10 (NatA) or E) NAA20 (NatB). Radiograph of immunoprecipated (IP) CFTR from cell lysates pulse-labeled for the time indicated in F) control or after NAA10 knockdown and G) control or after or NAA20 knockdown. Quantification and statistical analysis (n = 3, +/- SEM) presented for experiments from panel F) and G) in H.

Whereas knockdown of neither NatA nor NatB significantly altered degradation rates, the possibility that they altered steady state CFTR levels via effects on transcription or mRNA stability, CFTR mRNA levels were determined (Fig 6). NAA20, NatB, reduction had no effect on G85E or Q2P/G85E CFTR mRNA levels (Fig 6B and 6E). By contrast, NatA reduction leads to an increase in the levels of both G85E and Q2P/G85E CFTR mRNA of approximately 50% (Fig 6A and 6D) consistent with the increase in protein levels. The increase in mRNA levels suggests that NatA regulates CFTR through pathways involving mRNA production (transcription) or stability. These results demonstrate that NatA and NatB regulate CFTR by distinct mechanisms, NatA through control of mRNA and NatB through an alternative mechanism independent of changes in mRNA or protein degradation rates.

### Translation Rates of G85E CFTR

The translation rate of G85E CFTR was examined to determine 1) whether an increase in translation of CFTR upon NatA knockdown in correlation with the increase in mRNA and 2) whether, in the absence of changes in protein degradation and message levels, NatB regulates CFTR levels through alterations in the translation rate (Fig 6F–6H). Pulse experiments were completed under control and NatA or NatB knockdown conditions (Fig 6F–6H). As expected, due to the increase in mRNA levels upon NatA knockdown correlate with increased translation rates of G85E CFTR (Fig 6F and 6H). Knockdown of NatB had no effect on the translation rate of G85E CFTR (Fig 6G and 6H). Thus, regulation of G85E CFTR protein levels by NatB is occurring neither through alteration of protein degradation or protein production suggesting it may be regulating the conformational maturation of CFTR through modification of another protein.

### Mechanisms of N-Terminal Acetyltransferase Regulation of G85E CFTR Expression

**Fig 7:**
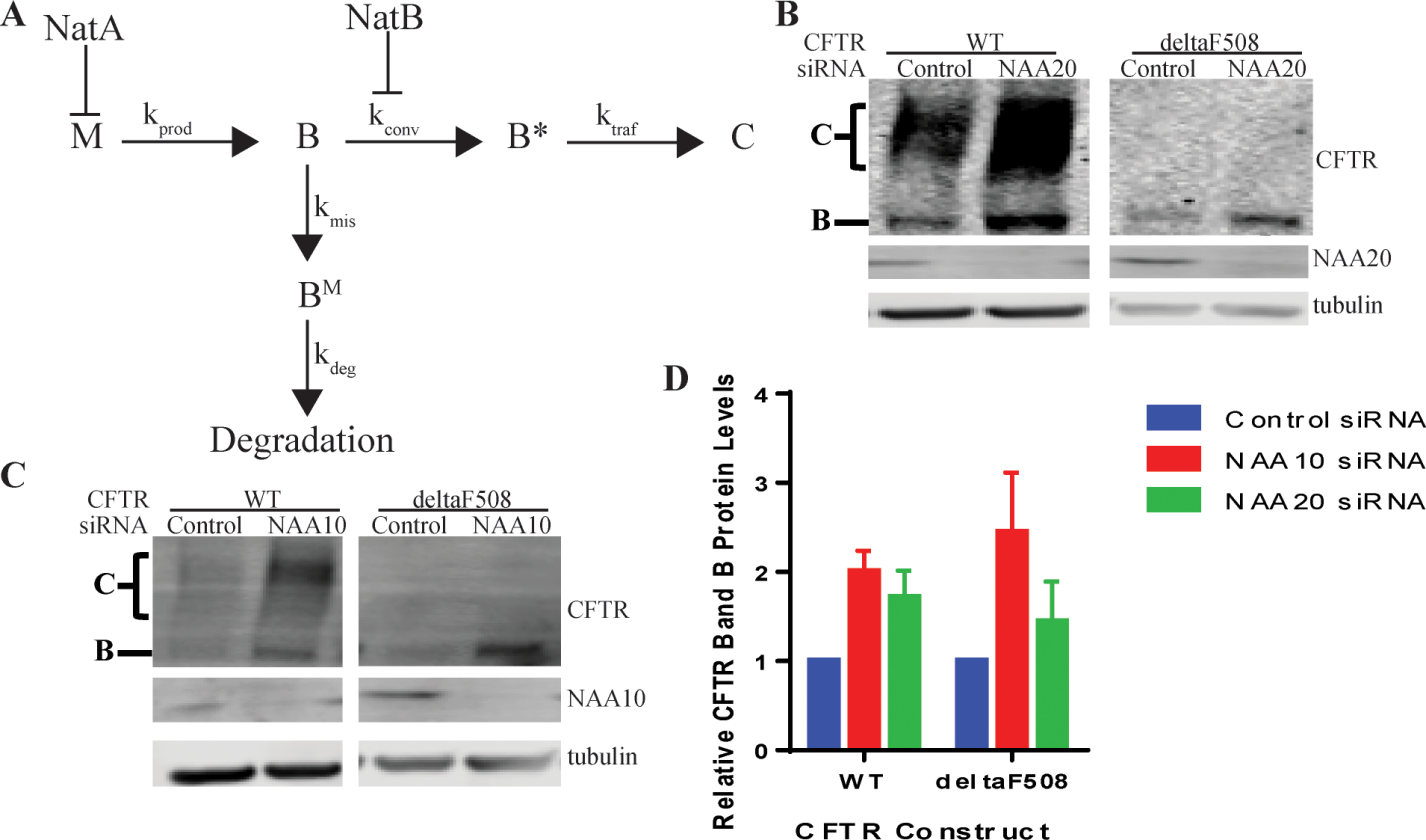
Schematic of Steps in CFTR Conformational Maturation and Points of Regulation by NatA and NatB. Schematic diagram for the regulation of CFTR expression by NatA and NatB (A) where M = amount of mRNA, B = ER retained CFTR sensitive to proteasome, B* = ER retained CFTR insensitive to proteasome, C = trafficked CFTR, k_prod_ = rate of production of B, k_deg_ = rate of degradation of B, k_conv_ = rate of conversion from B to B*, k_traf_ = rate of trafficking of B* to C, B^M^ = ER retained CFTR sensitive to proteasome, k_mis_ = rate of misfolding of B. Representative Western blots after SDS PAGE of steady state protein levels of WT and deltaF508 CFTR in cultured HeLa-Tet-On cells upon depletion of C) NAA10 (NatA) or B) NAA20 (NatB). Actin or tubulin are loading controls. D) Quantitation by LiCor and statistics (n = 3, +/- SEM).

The CFTR gene is transcribed and spliced to mRNA (M) and then translated to protein (Fig 7). The CFTR protein is cotranslationally integrated into the ER membrane where it is core glycosylated (B). The majority of the B form (70 to 80% of wildtype in heterologous expression [2] and much greater than 95% for Class 2 mutants [3]) becomes misfolded, B^M^ and is degraded by the proteasome, while a small portion of wild type B is converted to a proteasome resistant and export competent form (B*) in a poorly understood step [2] (Fig 7). This conversion is dependent on ATP [2] (Fig 7). B* can then be trafficked through the secretory pathway to the Golgi by COPII, where it becomes complex glycosylated, before proceeding to the plasma membrane (C) [2] (Fig 7). A simplified schematic for these reactions and the attendant rate constants associated with each step is presented in Fig 7. From the results presented above, it can be concluded that NatA regulates CFTR through inhibition of the production of mRNA (M) (Fig 7). This increase in M is reflected in an increase in total band B ([B]^total^) (Fig 7). Similar increases in CFTR protein levels were observed for both WT and deltaF508 upon depletion of NAA10 (NatA) (Fig 7C and 7D).

NattB, on the other hand, neither affects mRNA levels (M), translation rate (k_prod_), nor degradation rate (k_deg_); and, since, G85E is a severe mistrafficking mutation neither C nor rate of trafficking (k_traf_) are changed (Fig 7). Since B and B* cannot be differentiated by SDS PAGE, by deduction the increased steady state levels are predicted to be due to a change in B* levels through an increase in the rate of conversion (k_conv_) upon knockdown of NatB (Fig 7). The increase in conversion efficiency increases the amount of proteasome insensitive B* while decreasing the amount of B available for degradation. This change in B increases the total amount of [B]^total^ (Fig 7) as experimentally observed in Western blots (Figs 3C and 7B).

Despite this increase in ER export competent, B*, CFTR, the G85E mutation still prevents proper trafficking of CFTR to the plasma membrane possibly through impaired interactions with the COPII machinery. This prevention of trafficking, however, does not lead to degradation of the ER export competent forms [2, 6]. To further test the model of NatB’s inhibition of the formation of B*, both WT and deltaF508 CFTR protein levels were examined upon depletion of NatB (NAA20) (Fig 7B). As with G85E CFTR, deltaF508 CFTR only increased in ER retained CFTR, B (Fig 7B) further indicating that the increase in B* is not sufficient for productive interactions with COPII. By contrast, WT CFTR protein levels increases for both the ER retained, B, and trafficked CFTR, C. The WT CFTR results indicate that depletion of NatB increases B* and, when B* is capable of interacting with the COPII machinery, trafficked CFTR, C, is also increased thereby supporting the model of NatB’s involvement in the conversion of ER retained protein to a ER export competent and proteasome insensitive form, B*.

## Discussion

Acetylation has many and varied roles in the cell, thus it is not surprising that the activity of two of the complexes affect CFTR protein expression. The data presented in this study, at least in the case of CFTR, falsify the recent hypothesis that differential acetylation of mutant proteins fill a unique quality control role by acting as a signal for ubiquitinylation and degradation [9]. This hypothesis was particularly attractive in in light of the preferential proximity of a component of one of the transferase complexes to the nascent chain of a class 2 CFTR mutation (Fig 1). The effect of the complexes NatA (NAA15/NAA10), NatB (NAA25/NAA20), and NatE (NAA15/NAA10/NAA50) were first assessed utilizing siRNA knockdown. The findings demonstrated that some but not all of the complexes influenced CFTR expression. NatA, consisting of NAA15 and NAA10, and NatB, consisting of NAA20 and NAA25, but not NAA16 nor NatE, inhibit the expression of G85E CFTR, as well as the WT and deltaF508 variants (Fig 7 and S1 Fig). The mechanism of regulation employed by the two complexes is distinct, with NatA activity reducing mRNA levels and thereby the translation rate of G85E CFTR, and NatB inhibiting the conversion of the ER retained protein ([B]^total^) from a proteasome sensitive (B) form to a proteasome insensitive form (B*) (Fig 7).

The Q2P mutant, a mutation that is inhibitory of all known N-terminal acetylation reactions [12, 13], results indicate that CFTR is not a substrate for the enzymes. The Q2P/G85E CFTR protein behaved in a similar manner to the normal G85E CFTR protein under steady state conditions and after knockdown of either NatA or NatB (Fig 4). These results, as well as unpublished data with other Q2P CFTR mutants, are inconsistent with direct acetylation of the N-terminus of CFTR by NatA, NatB or any other known N-terminal acetyltransferases. The effects of NatA must therefore occur through involvement of an intermediate protein in the either the transcription of CFTR mRNA levels, possibly RNA polymerase II or other known gene silencing proteins [14]. The regulation of NatA is most likely a more general mechanism affecting many other mRNAs.

Knockdown of NatB did not influence mRNA levels, translation rates, or degradation rates, ruling out these mechanisms involvement in the increase in steady state levels of protein. Since G85E CFTR is a mistrafficking mutation and reduction in NatB levels only increases the ER retained protein, [B]^total^, and not the trafficking of the mutant, our hypothesis is that acetylation affects the conversion of B to B* (Fig 7). Very little literature is written about the conversion from B to B* [3]. What is known is that for heterologously expressed WT CFTR the majority of B is degraded quickly (B), while only a small portion (B*) is ultimately trafficked to the plasma membrane [3]. While B is sensitive to proteasome degradation, B* is in a conformation that is insensitive to the degradation and competent to be exported by the COPII machinery [3, 4, 6]. The conversion of B to B* occurs after the synthesis of B and requires ATP [2]. However, once conversion is complete, B* remains in this form, which is no longer dependent on ATP, and can then be trafficked [3]. It has been demonstrated that Class II mutants affect trafficking of CFTR to the plasma membrane (C) through the posttranslational folding of B and not due to changes in translation rate or degradation rate of B [15] suggesting that, in at least some cases, the mutation can inhibit a step subsequent to the formation of the proteasome insensitive B*. Whereas these two forms, B and B*, run at the same molecular weight on a SDS-PAGE gel, they cannot be distinguished using currently available biochemical methods [3].

CFTR destined for degradation (B form) is localized in a different part of the ER called the penultimate station, which is where ER transmembrane proteins destined for degradation are localized with the necessary machinery for translocation and degradation [5]. COPII cargo destined for the plasma membrane, such as wild type CFTR (presumably B*), is localized in another specialized compartment of the ER called vesicular tubular clusters (VTCs), or ER exit sties, similar to another transmembrane protein VSV-G [16]. VTCs contain all the necessary chaperones for trafficking out of the ER. Recruitment of CFTR to the VTCs likely prevents access of CFTR to ERAD proteins [3, 7]. Notably, if COPII-dependent trafficking is inhibited, CFTR remains in the ER but does not become a substrate of ERAD because it is not in the penultimate station [3, 7].

Despite the evidence for two forms of ER resident CFTR, the mechanism behind the conversion between the two forms remains a mystery twenty years after its initial discovery. Studies have shown that the formation of B* does not required complex glycosylation or trafficking from the ER, indicating that the formation of B* occurs before the Golgi and formation of C [3]. By depleting ATP before folding of CFTR, the B* form fails to form and the formation of C is inhibited [3]. Interaction with chaperones may play a critical role in the B to B* conversion. Other studies have shown that Hsp90 is important for the proper trafficking of CFTR [17]. In addition, Hsp70 binds both mutant and WT CFTR but dissociates from WT before trafficking can occur, while remaining bound to mutant CFTR [18].

The data presented in this study provide new insight into this conversion mechanism, suggesting it is regulated by N-terminal acetylation of a protein other than CFTR itself. Determining the identity of the target of the modification required for the conversion is the critical next step. Identification of new players in the conversion from B to B* may provide an entrez to a more direct study into the other proteins involved in the critically relevant conversion mechanism and the role of acetylation in protein maturation.

## Materials and Methods

### *In Vitro* Cotranslational Photocrosslinking

Photocrosslinking experiments were performed as previously described [11, 19]. Photocrosslinking probe was positioned in codon 93 of CFTR. The amber-mutation was introduced in that position by site-directed mutagenesis and mRNAs were synthesized in *in vitro* transcription system using SP6 RNA polymerase. Modified amber-suppressor tRNAs were used for introduction of the photocrosslinking probe ANB in *in vitro* translation system as described [11]. To make stable-ribosome nascent complexes, the mRNAs were truncated at codon 156.

### Identification of Proteins Interacting with CFTR Nascent Chains during Translation

For identification of proteins interacting with nascent chains during translation the approach similar to the published earlier iPINCH was used [11]. Standard wheat germ extract (similar to the extract used for *in vitro* translation) was used for fractionation in these experiments. Lysate (25 mL) was centrifuged at 270,000 g for two hours. Pellet was solubilized in buffer containing 50 mM HEPES (pH 7.5), 450 mM potassium acetate (KAcetate), 5 mM MgCl_2_, 2 mM glutathione and incubated for 30 min, centrifuged at 540,000 g for 2 hours. Supernatant was dialyzed against buffer containing 25 mM HEPES (pH 7.5), 40 mM KAcetate, 2 mM glutathione. The containing ribosome-associate proteins solution was used for fractionation by FPLC. Two independent schemes for fractionation were used: first scheme included Heparin column fractionation first, then MonoQ; the second scheme used MonoQ column first and Heparin after that (see Fig 2 and S1 Fig for details). Fractions were tested for the presence of proteins forming a photoadduct with CFTR nascent chains similar to iPINCH approach described earlier [11]. The fractions with a strong crosslink to a ~100 kDa protein were selected. The proteins were separated by SDS-PAGE and the ~100 kDa bands were excised from the gels. Mass spectrometry was conducted by the Protein Chemistry Core Facility, UT Southwestern Medical Center as described [11]. The resulting files were searched against NCBI-nr protein sequence databases through the Mascot search engine (Matrix Science).

### Plasmids, DNA Techniques

Full length WT CFTR in the pBI-CMV2 expression vector, a gift from J. Rommens, (The Hospital for Sick Children, Toronto) was the parent for all mutations which were introduced using standard protocols [20]. PCR site-directed mutagenesis was performed using PfuUltra high-fidelity DNA Polymerase (Agilent) and mutations were confirmed by DNA sequencing. G85E was introduced into the WT CFTR construct, while the Q2P was introduced into the G85E CFTR construct. The sense primers used are listed in Table 1.

**Table 1:** Sense Primers for Generation of DNA Constructs.

Primer (5'-3' sense primers)	Mutation
CCAGCGCCCGAGAGACCATGCCGAGGTCGCCTCTGGAAAAG	Q2P
GAGATTTATGTTCTATGAGATCTTTTTATATTTAGG	G85E

### Cell Culture and Transfection

All experiments were conducted using HeLa Tet-On cells (Clonetech) maintained at 37°C, 5% CO_2_, and in Dulbecco’s modified eagle’s medium (DMEM) 11965 (Invitrogen) with 10% fetal bovine serum (FBS) (Hyclone), and 1% penicillin/streptomycin (GIBCO). Either Silencer Negative Control #2 siRNA (Ambion) or siRNA synthesized by Dharmacon (Table 2) was transfection using RNAiMax reagent (Invitrogen) to a final concentration of 2nM and incubated for 48 hours. Negative control #1 had off target effects that made it inappropriate for these studies. Cells were transfected with 3 μg of plasmid using Lipofectamine 2000 reagent (Invitrogen) and grown for 24 hours. Cells were washed with phosphate buffer saline (PBS) pH 7.4 and lysed at 4°C for 1 hour in RIPA buffer (20mM tris pH 7.6, 150mM NaCl, 0.1% SDS, 1% IGEPAL, 0.5% deoxycholic acid, 1 mg EDTA-free protease inhibitor tablet (Roche)). Soluble and insoluble fractions were separated by 13000g centrifugation for 20 minutes. The insoluble (pellet) fraction was discarded, while soluble (supernatant) fraction was solubilized in sample buffer (60mM tris pH 6.8, 5% glycerol, 2% SDS, bromophenol blue, 280 mM β-mercaptoethanol) and incubated at 37°C for 20 minutes prior to SDS PAGE.

**Table 2:** siRNA Sequences for Knockdown.

siRNA sequence (5'-3' sense)	Protein
Pool of four: GCUGAAAGAUUAUGAUAUG, CAAAUUAGCACUUUAGAAG, CGAAAUGCCCUCAAAUUAG, GUUCGUAAAGGACUUCGUA	NAA16
GAUAUGAAGAUCACAGUUA	NAA15
CCAGAUGAAAUACUACUUCUU	NAA10
Pool of four: GCAGAAGGCUCAGUAGCUA, GAAAUCCAUCAUACCAUUA, UAACUUGGAUCCACUUACA, GUGGAGAAUUAAUGGGUUA	NAA20
Pool of four: CGGAGAAGAUGCCGAGAA, GAGACAGGAAAGCGAUUUA, GAAUAUCUGUGUACGCAAA, UGGGAAGAUUUGCGAGACA	NAA25
AUCCCUUGUAGAAUUGUCAUU	NAA50

### Western Blot Analyses

A 6%/10% (w/v) discontinuous polyacrylamide gel in a tris-glycine buffering system was use to separate cell lysate proteins prior to transfer to PVDF Immobilon-FL membranes (Millipore). Primary CFTR mouse monoclonal antibody 596 (UNC School of Medicine) or rat monoclonal 3G11 (www.CFTRfolding.org), ARD1 (NAA10) (Sigma-Aldrich), NARG1 (NAA15) (Bethyl Laboratories, Inc.), NAT-13 (NAA50) (Santa Cruz Biotechnology, Inc), NAT-5 (NAA20) (Santa Cruz Biotechnology, Inc), C12ofr30 (NAA25) (Thermo Scientific), actin (Millipore), β-tubulin (Sigma-Aldrich), secondary antibody IRDye 800CW or 698CW Goat (polyclonal) anti-mouse/rabbit/rat IgG (H+L) (LI-COR) were used for Western blotting. Western blots imaged on Odyssey CLx Infrared Imaging System (LI-COR). Quantification of bands was performed using ImageStudio Lite 4.0 (LI-COR).

### Pulse-Chase and Pulse Assay

HeLa Tet-On cells were siRNA and plasmid transfected as described above. Twenty-three hours after transfection, cells were starved for 30 minutes with DMEM media without methionine/cysteine (Invitrogen) then labelled with EasyTag EXPRESS ^35^S Protein Labelling Mix (100 μCi/ml, Perkin-Elmer) for a 30 minute pulse, washed with regular DMEM (with methionine/cysteine), and chased for either 0, 0.5, 1, 2, or 4 hours. After chase, soluble fractions were collected as described above.

A 350μL aliquot of the soluble fraction was immunoprecipated (IP) with mouse monoclonal CFTR antibody 7D12 (www.CFTRfolding.org). The remainder of the aliquot had sample buffer added and was analyzed by Western blotting (described above). Prior to IP of CFTR samples were precleared by rocking at 4°C for 30 minutes with 30μL Protein G agarose beads (Roche). Beads were pelleted, supernatant removed to new tube along with 7μL 7D12 (CFTR antibody, www.CFTRfolding.org), and rocked at 4°C for 1 hour. 30μL Protein G beads were added and rocked for 20 to 24 hours at 4°C. Beads were pelleted, supernatant discarded, beads washed three times with RIPA buffer, resuspended in sample buffer, and analyzed by SDS-PAGE gel. Protein separated by SDS-PAGE were transferred from the gel to PDVF Immobolin-FL membranes and subjected to phosphor imaging and analyses using a Typhoon 9410 Variable Mode Imager (GE Healthcare). Quantification was performed utilizing ImageStudio Lite Version 4.0 (LI-COR) and signals were normalized using CFTR Western blot of the IP at chase time zero. The percent remaining of CFTR was calculated relative to G85E CFTR at chase time zero and plotted as a function of chase time to generate degradation curves.

For pulse experiments, the labeling was performed as above without removal of the label and subsequent chase. Quantification was performed by ImageStudio Lite Version 4.0 (LI-COR) and samples normalized to CFTR Western blot of IP. Relative quantity of radiolabeled CFTR was calculated by dividing radiolabeled CFTR to the amount of radiolabeled CFTR at the 30 minutes pulse under control siRNA conditions.

### cDNAs, Quantitative Real Time-PCR

NucleoSpin RNA II kit (Clontech) was used to purify total RNA. cDNAs were synthesized using High-Capacity cDNA reverse transcription kit (Applied Biosystems). cDNAs analyzed by qPCR utilizing Power SYBR Green and the 7900T Fast Real-Time PCR System (Applied Biosystems). The following primers were used to detect RNA levels of CFTR (1–2), GFP, (3–4), Actin (5–6), NAA10 (7–8), NAA15 (9–10), NAA20 (11–12), and NAA25 (13–14) (see Table 3). qPCR data was analyzed using the comparative C_T_ method [21].

**Table 3:** Primers for qPCR Analysis.

Primer (5'-3')	mRNA	Number
GTGGATGCTGTTGTCTTTCGG	CFTR	1
AGGCACGAAGGAGGCAGTC	CFTR	2
GAAGTAGATCATGTGATCGCG	GFP	3
CTGCTGCCCGATAACCACT	GFP	4
CACCTTCTACAATGAGCTGCG	Actin	5
TAGCACAGCCTGGATAGCAAC	Actin	6
ATCAGTTTCTGAGCCAGACCG	NAA10	7
GAAGAGGACCCAGATGATGTGC	NAA10	8
CGAGATCTTGAGGGTACAGGGAAACGAG	NAA15	9
GCATAACCAATCCATGATGCTCTCTGC	NAA15	10
CGAGGATCGGAAGCTGGTCA	NAA20	11
CCACTGCCCAGGGTTATTCA	NAA20	12
TCCTCAGGGACAGTGTGTGA	NAA25	13
GTAGGAAGTTTCAATAACAAGCCT	NAA25	14

## Supporting Information

S1 Fig Alternative Scheme for the Identification of Proteins Interacting with CFTR Nascent Chains During Translation. Ribosome-associated proteins were released from wheat germ ribosomes by high salt treatment and were subjected to dialysis. Proteins were fractionated by FPLC on MonoQ column (A), fractions were tested by crosslinking analysis (C), and selected fractions were fractionated by FPLC on Heparin column (D), were tested by crosslinking analysis again (F), selected fraction was separated by SDS-PAGE (G), and protein band of appropriate molecular weight was excised from the gel and used in mass spec. A, FPLC chromatography profile (MonoQ); B, Electrophoresis of fractions (Coomassie); C, Test by photocrosslinking (autoradiography); D, Second FPLC (Heparin); E, Electrophoresis of fractions (Coomassie); F, Fraction analysis as in C; G, Electrophoresis and Protein ID by Mass Spectrometrty.
(TIFF)

S1 Table Identification of Proteins Interacting with CFTR Nascent Chains During Translation by Mass Spectrometry. (XLSX)
