# Supplementary figures and images for: N-Alpha-Acetyltransferases and Regulation of CFTR Expression
Source: PLoS One. 2016 May 16;11(5):e0155430. doi: 10.1371/journal.pone.0155430 (PMC4868295; doi:10.1371/journal.pone.0155430)

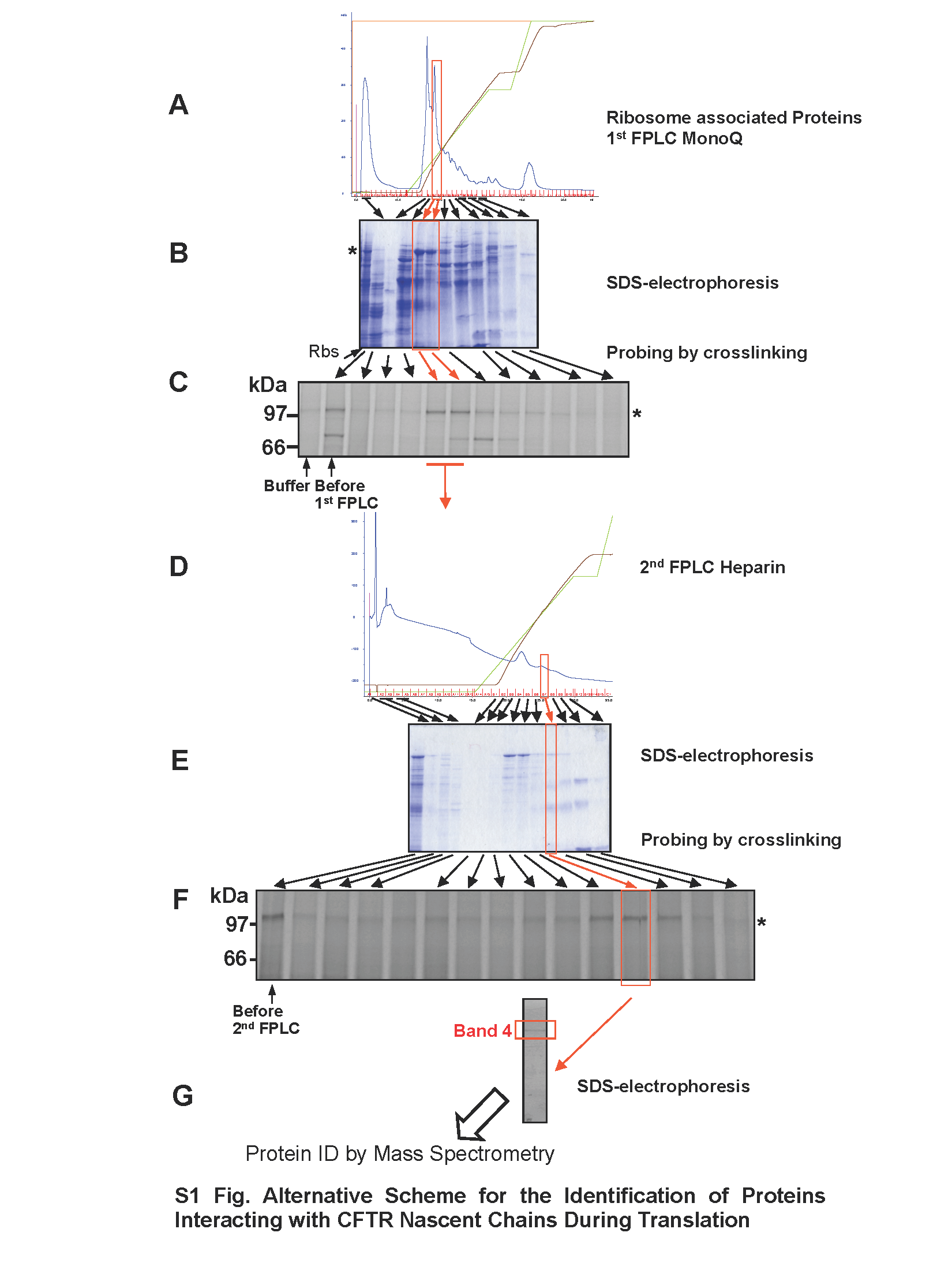

Supplement: S1 Fig — Ribosome-associated proteins were released from wheat germ ribosomes by high salt treatment and were subjected to dialysis. Proteins were fractionated by FPLC on MonoQ column (A), fractions were tested by crosslinking analysis (C), and selected fractions were fractionated by FPLC on Heparin column (D), were tested by crosslinking analysis again (F), selected fraction was separated by SDS-PAGE (G), and protein band of appropriate molecular weight was excised from the gel and used in mass spec. A, FPLC chromatography profile (MonoQ); B, Electrophoresis of fractions (Coomassie); C, Test by photocrosslinking (autoradiography); D, Second FPLC (Heparin); E, Electrophoresis of fractions (Coomassie); F, Fraction analysis as in C; G, Electrophoresis and Protein ID by Mass Spectrometrty. (TIFF) [file pone.0155430.s001.tiff]
